# Impact of neighborhood context on self-rated health among very old adults living in Germany: a cross-sectional representative study

**DOI:** 10.1186/s12877-024-05175-y

**Published:** 2024-07-05

**Authors:** Jaroslava Zimmermann

**Affiliations:** Cologne Center for Ethics, Rights, Economics, and Social Science of Health (ceres), University of Cologne, Albertus- Magnus-Platz, 50923 Cologne, Germany

**Keywords:** Oldest old, Physical environment, Socioeconomic deprivation, Social cohesion, Place attachment, Walkability, Germany

## Abstract

**Background:**

Increasingly, evidence has shown that different aspects of neighborhood context play a significant role in self-rated health, one of the key health indicators in advanced age. Nevertheless, very old adults are often under represented or excluded from such research. Therefore, the first aim of this study was to examine whether social, socioeconomic, and physical neighborhood context is associated with self-rated health in the very old population of Germany. The second objective was to explore whether the link of socioeconomic and physical neighborhood context with self-rated health is moderated by availability of social resources in neighborhoods.

**Methods:**

Data from the representative survey, “Old Age in Germany” (D80+) were employed. In total, the study sample of D80+ included 10,578 individuals aged 80 years and over. Additionally, the D80+ data were matched with the freely accessible regional dataset of the Federal Institute for Research on Building, Urban Affairs, and Spatial Development. Two self-rated items (place attachment and social cohesion) were used to assess social neighborhood context. Socioeconomic context of neighborhoods was operationalized by German index of socioeconomic deprivation. To evaluate physical context, perceived measures of building conditions and walkability were included. Using the maximum likelihood estimator with robust standard errors, logistic regression models were estimated to analyze the relationship between neighborhood context (social, socioeconomic, and physical context, as well as their interactions) and self-rated health.

**Results:**

Including 8,066 participants in the analysis, the findings showed that better condition of residential building, higher walkability, being closely attached to outdoor places, and higher social cohesion were associated with higher chance to report good self-rated health of very old adults. In the adjusted models, the German socioeconomic deprivation index was not related to self-rated health. The effect of socioeconomic and physical neighborhood context on self-rated health did not differ according available neighborhood social resources.

**Conclusions:**

The results indicate that especially more favorable conditions in social and physical neighborhood context are associated with good self-rated health in the very old population of Germany. Further studies should consider multiple aspects of neighborhood context as well as their interplay when examining the neighborhood impact on self-rated health in older populations.

**Supplementary Information:**

The online version contains supplementary material available at 10.1186/s12877-024-05175-y.

## Background

Many industrialized countries face the challenge of demographic change, leading to shifts in the population age structure. In Germany, very old adults constitute the fastest-growing population group [1]. In 2021, 7% of Germany’s inhabitants were aged 80 years or older, and this proportion is expected to double over the next few years [1]. In gerontology, the age of over 80 years is often described as “Fourth Age” which, in contrast to the more active and healthy age group of 60 to 80 years, is more frequently characterized by frailty and health limitations [2]. Environmental gerontology [3] proposes that individual quality of life in advanced age increasingly depends on how person’s competencies match the requirements of her or his environment. Particularly due to increasing health impairments, very old adults spend most of their time at home or in their immediate living environment [4]. Moreover, very older adults often live in the same place for a long time and consequently they develop strong ties to the place [5]. Therefore, home and neighborhood contexts gain on relevance especially in the oldest age [6]. Considering health status as one of the major outcomes of person-environment linkage, Wahl and Gerstorf [6] demonstrated that various aspects of environmental contexts (e.g., social, socioeconomic, physical) may affect individual health. At the same time, the authors highlighted that proximal contexts with rather direct effect (e.g., own house or apartment) as well as distal contexts with rather indirect effect (e.g., neighborhood) may considerably shape health status during the life span [6].

Owing to its high predictive power for adverse health outcomes (e.g., mortality or healthcare utilization), self-rated health (the subjective evaluation of one’s own physical and mental health status) is one of the most relevant health indicators in old age [7–10]. The evidence has confirmed that especially social (e.g., social cohesion) and socioeconomic contexts (e.g., average income level) of neighborhoods are associated with self-rated health among older adults [11–15]. Neighborhood physical context has been rarely examined with regard to self-rated health in older age and the few existing studies showed inconclusive findings [16, 17]. Nevertheless, these studies are based on small sample sizes and cover small geographical areas (e.g., a town or municipality). An umbrella review [18] focusing on physical activity in older age – which is an important predictor of self-rated health [11, 19–21] – confirmed that, for instance, low walkability, poor street lighting or lack of aesthetically pleasing scenery in neighborhoods were associated with low physical activity among older adults.

The majority of studies investigating the link between neighborhood context and self-rated health in older age consider one aspect of neighborhood context, such as social [11, 15], socioeconomic [14], or physical context [16, 17]. Wahl and Gerstorf [6] demonstrated that focusing on a single contextual aspect may ignore potentially important sources for explaining variance of individual outcomes. I could identify two recent studies considering simultaneous effects of two aspects of neighborhood context (social and socioeconomic context) on self-rated health [12, 13]. The findings of the English Longitudinal Study of Aging [12] confirm that both, neighborhood socioeconomic deprivation (computed as an index based on objective neighborhood characteristics, such as income level, employment, and crime rate) and higher neighborhood dissatisfaction (using a summed score of nine items, e.g., sense of belonging to the area, trustworthiness, and safety) are associated with poor self-rated health; this association did not change over time. Using cross-sectional data, Stroope et al. [13] identified no association between the neighborhood economic disadvantage index (including unemployment rate and poverty level) and self-rated health. However, older adults with more positive ratings of the neighborhood social environment (using index-based aspects, e.g., perceptions of community attachment, animosity, or social ties) were more likely to report satisfactory self-rated health [13].

Nonetheless, in most of the aforementioned studies [11, 12, 14–17], middle-aged (40 years or older) or younger older adults (60 years or older) are included whereas very old population (e.g., aged 80 years or older) remains underrepresented. There is evidence suggesting that neighborhood impacts on health may differ among the age groups [22]. Abe et al. [22] reported, for instance, that higher social cohesion was associated with lower odds being frail in older adults (65 years or older) while there was no effect in middle-aged adults (50 to 65 years). This finding supports the assumption of environmental gerontology [3] assuming that the impact of environment intensifies with increasing age. Until now, little is known how neighborhood context influences self-rated health in the oldest age. Moreover, to the best of my knowledge, there are no other studies investigating the simultaneous effects of social, socioeconomic, and physical neighborhood context on self-rated health in older age. Hence, the first aim of the current study was to examine whether diverse aspects of neighborhood context (i.e., socioeconomic, social, and physical context) are associated with self-rated health in the very old population of Germany.

Furthermore, proponents of social disorganization theory demonstrated that disadvantaged neighborhoods (e.g., with high poverty rate) often lack social ties and mutual trust between neighbors (e.g., social cohesion) [23, 24]. Social ties in neighborhoods can be viewed as resources providing information, emotions, or material goods that support adaptation to different external stressors (e.g., low walkability of outdoor place because of neglected sidewalks), which might help to prevent adverse health responses [25, 26]. Considering the fact that very old adults experience functional and cognitive limitations much more frequently than any other age groups, they are particularly vulnerable to environmental stressors [6]. Applying the environmental docility hypothesis [27], lack of resources, such as social relationships in neighborhoods or emotional attachment to place, which may help to compensate for or to adapt to unfavorable environmental condition may lead to deterioration of health among very old adults in the long-term. Thus, it can be hypothesized that very old adults who live in neighborhoods with poor physical and/or socioeconomic condition and lack social resources in neighborhoods (e.g., low social cohesion) will more likely experience poor self-rated health than those living in neighborhoods with available social resources. To my best knowledge, this hypothesis has not yet been explored in older populations. Therefore, the second aim of the current study was to examine whether the associations of socioeconomic and physical neighborhood context with self-rated health of very old adults differ according to the availability of social resources in neighborhoods.

## Methods

### Study design and data description

The study employed data collected between November 2020 and April 2021 from the German representative study “Old Age in Germany” (D80+) (Clinical Trial Number: DRKS00017706). Data were collected during the second and third waves of the COVID-19 pandemic, which were characterized by the highest infection and death rates in the oldest population groups in Germany [28]. The study sample included individuals living in private households and nursing homes, who reached the age of 80 years on or before March 1, 2020. A multi-stage sampling procedure was undertaken. First, 461 municipalities in Germany were randomly selected. Second, a random sample of residents was drawn from the population registers of the selected municipalities. The gross sample size was calculated based on a random sample (*N* = 40,209). In total, 10,578 individuals participated in the study through written questionnaires (*N* = 10,360) or computer-assisted telephone interviews (*N* = 218). Most telephone interviews were conducted with a proxy person (*N* = 193) because of the considerable health impairment of the target individual. In all analyses, data weights were applied to correct for disproportionate sampling design (e.g., oversampling of men and individuals aged 85 years or older), non-availability of phone numbers, and non-response in the survey.

Additionally, freely accessible regional data from the INKAR (Indicators and Maps on Spatial and Urban Monitoring) database of the Federal Institute for Research on Building, Urban Affairs and Spatial Development were employed. In the INKAR database, municipality is the smallest available geographical unit. Using official municipal codes, the selected regional characteristics of the municipalities described below were matched with the anonymized D80+ dataset. Considering the current addresses of the target individuals that were provided during data collection, the final sample included participants from 526 municipalities in Germany.

### Measures

#### Dependent variable

Within D80+, the self-rated health status was measured using one item from the Short-Form Health Survey (SF-8) [29]. The participants were asked to rate their overall health status in the last four weeks on a 4-point Likert scale, ranging from very poor to very good. As explained in the statistical analyses section, the measure of self-rated health was dichotomized into poor (very poor/rather poor) versus good (rather good/very good) health status.

#### Independent variables

To operationalize social context of neighborhood, two self-rated items from D80+ were included: attachment with the place and social cohesion. To measure *place attachment*, participants were asked, “How closely do you feel connected to your living environment?,” with four responses options (ranging from ‘not close at all’ to ‘very close’). One item was used to assess *social cohesion*: “Can you trust people in your neighborhood?” The response categories ranged from ‘strongly disagree’ to ‘strongly agree’ on a 5-point Likert scale.

Physical neighborhood context was operationalized by two self-rated items collected in D80+, namely, building condition and walkability of external living environment. Participants evaluated the *condition of the house or nursing facility* where they lived, on a 3-point Likert scale ranging from ‘very good’ to ‘requiring renovation.’ *Walkability* was assessed with the question “How suitable is your external living environment for walking, using wheelchair, or managing things?,” with four answer categories ranging from ‘not suitable at all’ to ‘very suitable.’

Socioeconomic context of neighborhood was measured by *German Index of Socioeconomic Deprivation* (*GISD*) using data from the INKAR database. The multidimensional GISD was constructed by Michalski et al. [30] using principal component analysis. GISD consists of three equally weighted dimensions: education, employment, and income. In each dimension, three objectively measured indicators of socioeconomic deprivation were included. The educational dimension included the proportion of school drop-outs without qualification, employees without qualification, and employees with university degrees. The employment dimension considers the unemployment rate, employment rate, gross wages, and salaries. The income dimension includes debtor quota, net household income, and tax revenue. The GISD values range from 0 (lowest degree of deprivation) to 1 (highest degree of deprivation). For the current analysis, the most recently available data for GISD were used, referring to the territorial status as of 31st December 2019. Data from the smallest available regional unit, i.e., the municipal level, were included. Further details regarding GISD have been published elsewhere [30]. The municipalities included in the current analysis comprised of an average of 320,827 inhabitants (range: 327–3,664,088). For comparison, there were 10,799 municipalities in Germany, with an average of 7,701 inhabitants (range: 10–3,669,491) [30].

#### Control variables

From the D80+ data, the following control variables were included: *age* (in years), *gender*, *living alone* (no/yes), *type of residence* (private household/nursing home), *migration background* (no/yes), and *educational level*, based on the International Standard Classification of Education (ISCED) [31]. The ISCED scale was generated using information on school, vocational, and academic education. Following categories were distinguished: low (ISCED: 0 to 2), middle (ISCED: 3 to 4), and high (ISCED: 5 to 8). Additionally, the *region of residence* (East/West Germany) was considered because of the ongoing socioeconomic and health disparities between East and West Germany, based on different historical developments after the Second World War. The *municipality type* (urban/rural) was adopted from the INKAR dataset of the Federal Institute for Research on Building, Urban Affairs, and Spatial Development. Furthermore, the mean score for *instrumental activities of daily living* [32] and *number of self-reported medically treated chronic disease*s were considered relevant health characteristics. For further details, please refer to Table 1.

**Table 1:** Descriptive characteristics

Variables			Study sample (*N* = 10,578)	Final analytic sample (*N* = 8,066)
			% or mean (SD)	% or mean (SD)
*Dependent variable*				
Self-rated health status				
	poor		37.8	38.5
	good		60.1	61.5
	missing values (%)		2.1	-
*Independent variables*				
Place attachment (1 = not close at all; 4 = very close)			2.7 (1.0)	2.8 (1.0)
	missing values (%)		2.8	-
Social cohesion (1 = strongly disagree; 5 = strongly agree)			4.1 (1.1)	4.1 (1.1)
	missing values (%)		3.3	-
Building condition (1 = very good; 3 = requiring renovation)			1.5 (0.6)	1.5 (0.6)
	missing values (%)		3.1	-
Walkability (1 = not suitable at all; 4 = very suitable)			3.0 (0.8)	3.0 (0.8)
	missing values (%)		4.1	-
GISD (0 = low deprivation; 1 = high deprivation)			0.6 (0.2)	0.6 (0.2)
	missing values (%)		-	-
*Control variables*				
Age (years)			86.0 (4.1)	86.0 (4.1)
	missing values (%)		-	-
Gender				
	female		62.1	61.7
	male		37.9	38.3
	missing values (%)		-	-
Living alone				
	no		43.1	45.6
	yes		52.4	54.4
	missing values (%)		4.6	-
Type of residence				
	private household		89.8	90.1
	nursing home		10.2	9.9
	missing values (%)		-	-
Migration background				
	no		77.3	79.7
	yes		22.0	20.3
	missing values (%)		0.7	-
Education level				
	low		22.8	22.7
	middle		49.9	51.2
	high		24.1	26.1
	missing values (%)		3.2	-
Region of residence				
	East Germany		21.9	22.5
	West Germany		78.1	77.5
	missing values (%)		-	-
Municipality type				
	urban		90.2	90.3
	rural		9.8	9.7
	missing values (%)		-	-
IADL (0 = not possible without help; 2 = no help needed)			1.4 (0.7)	1.4 (0.7)
	missing values (%)		1.2	-
Number of chronic diseases			4.7 (2.7)	4.7 (2.7)
	missing values (%)		1.6	-

Note. Weighted data. SD, Standard Deviation; GISD, German Index of Socioeconomic Deprivation; IADL, Instrumental Activities of Daily Living

### Statistical analyses

The statistical analyses were conducted in four steps. Firstly, descriptive (Table 1) and correlational analyses (Supplement Table S1) were carried out. There was no indication of multicollinearity between the variables included in the analyses. Secondly, the testing of the proportional odds assumption using Brant-Wald test [33] showed that applying an ordinal logistic regression with the four-level outcome measure of self-rated health could lead to estimation bias. Therefore, in line with previous studies [11–13, 17], the measure of self-rated health was dichotomized differentiating between poor (zero) and good (one) self-reated health status. Thirdly, due to the hierarchical structure of the data, multilevel logistic regression models with random intercepts (using maximum likelihood estimator with robust standard errors and logit link function) were estimated defining municipalities as clusters. The intercept-only-model was estimated to calculate intraclass correlation coefficient (ICC) for multilevel logistic regression [34] indicating that only 3% (ICC = 0.03) of the chances experiencing good health status can be explained by the differences between the municipalities. The average cluster size was 15.85 participants per municipality. As demonstrated by Muthén & Satorra [35], low ICC and small average cluster size indicate that data clustering (e.g., estimating a within and between part of the model) does not have to be considered. In this case, an alternative single-level model can be applied adjusted for the non-independence of observations and unequal selection probability using sandwich estimator to compute robust standard errors [36, 37].

Subsequently, in the fourth step, single-level logistic regression models (using sandwich estimator to compute robust standard errors) were applied to examine the association between multiple aspects of neighborhood context and self-rated health. As explained above, all predictors were included on the individual level. To increase the interpretability of the moderation effects, continuous predictors were mean-centered. A series of four models were estimated. In Model 1, the independent variables were included. In Model 2, all control variables were added. Finally, the moderation effects of social neighborhood context were tested, including interaction terms of place attachment (Model 3a) and social cohesion (Model 3b), in separate analyses with each indicator of socioeconomic (GISD) and physical context (building condition, walkability). The final analysis sample was 8,066 including only participants with no missing values in included variables. Table 1 provides detailed information on missing values for each variable. Sensitivity analyses showed that replacing the missing values using multipe imputation leads to similar results (Supplement Table S3). All analyses were performed using Mplus 8.6 software (Muthen & Muthén, 1998–2017). Statistical significance was set at *p* < 0.05.

## Results

As shown in Table 1 and 61.5% of the study sample reported good self-rated health. The majority of participants reported being closely attached to their outdoor environment, with an average rating of 2.8, and trusting the people in their neighborhood, with an average rating of 4.1. Similarly, building condition and walkability were assigned positive ratings of, respectively, 1.5 and 3.0 on average. The average GISD was evaluated at 0.6, representing moderate socioeconomic deprivation.

Table 2 presents results of logistic regression analyses for predicting good self-rated health among the very old population in Germany. The results of the unadjusted Model 1 show that all independent variables significantly predicted self-rated health in very old adults. More specifically, greater connection with outdoor spaces was associated with increased odds to report good self-rated health by 7% (Odds Ratio (OR) = 1.07; 95% Confidence Interval (CI) = 1.01–1.13). Similarly, increasing ratings of social cohesion were linked with a 24% increase in odds of reporting good self-rated health (OR = 1.24; 95% CI = 1.18–1.30). Deteriorating building condition was associated with a 25% decrease in odds of reporting good self-rated health (OR = 0.75; 95% CI = 0.68–0.82). One unit increase in perceived walkability was associated with a 39% increase in odds of reporting good self-rated health (OR = 1.39; 95% CI = 1.31–1.48). Increase of municipality socioeconomic deprivation was associated with reduced odds of experiencing good self-rated health by 35% (OR = 0.65; 95% CI = 0.48–0.89).

**Table 2:** Results of logistic regression analyses predicting the likelihood of reporting good self-rated health in the very old population in Germany (*N* = 8,066)

Predictors	Model 1	Model 2	Model 3a	Model 3b
	OR (95% CI)	OR (95% CI)	OR (95% CI)	OR (95% CI)
*Independent variables*				
Place attachment	1.07* (1.01–1.13)	1.14*** (1.07–1.20)	1.14*** (1.07–1.20)	1.08 (0.93–1.26)
Social cohesion	1.24*** (1.18–1.30)	1.15*** (1.08–1.21)	1.14*** (1.08–1.21)	1.14*** (1.08–1.21)
Building condition	0.75*** (0.68–0.82)	0.81*** (0.73–0.91)	0.81** (0.73–0.92)	0.81*** (0.72–0.91)
Walkability	1.39*** (1.30–1.49)	1.19*** (1.10–1.28)	1.19*** (1.11–1.28)	1.19*** (1.10–1.28)
GISD	0.65** (0.48–0.89)	0.97 (0.64–1.48)	0.99 (0.65–1.50)	0.96 (0.63–1.46)
*Control variables*				
Age (years)		1.02* (1.01–1.04)	1.02* (1.01–1.04)	1.02* (1.01–1.04)
Gender (Ref. female)		1.04 (0.90–1.19)	1.04 (0.90–1.19)	1.03 (0.90–1.19)
Living alone (Ref. no)		1.04 (0.91–1.19)	1.04 (0.91–1.18)	1.04 (0.91–1.19)
Type of residence (Ref. private household)		2.44*** (1.76–3.39)	2.43*** (1.75–3.38)	2.47*** (1.78–3.44)
Migration background (Ref. no)		1.05 (0.90–1.23)	1.05 (0.90–1.23)	1.05 (0.90–1.23)
Education (Ref. low)				
middle		0.86 (0.72–1.02)	0.86 (0.72–1.02)	0.86 (0.72–1.02)
high		0.93 (0.77–1.13)	0.93 (0.77–1.13)	0.93 (0.77–1.13)
Region of residence (Ref. West Germany)		0.83* (0.69–0.99)	0.83* (0.69-1.00)	0.83* (0.69-1.00)
Municipality type (Ref. urban)		1.14 (0.91–1.43)	1.14 (0.91–1.42)	1.14 (0.91–1.42)
IADL		4.33*** (3.82–4.90)	4.33*** (3.81–4.91)	4.36*** (3.82–4.94)
Number of chronic diseases		0.76*** (0.75–0.78)	0.76*** (0.75–0.78)	0.76*** (0.75–0.78)
*Interaction terms*				
Building condition x place attachment			1.06 (0.96–1.18)	
Walkability x place attachment			1.01 (0.95–1.08)	
GISD x place attachment			1.15 (0.82–1.62)	
Building condition x social cohesion				1.04 (0.65–1.24)
Walkability x social cohesion				0.94 (0.88–1.01)
GISD x social cohesion				0.96 (0.63–1.46)
*Model fit*				
BIC	10,369.82	8,415.77	8,439.65	8,436.87
AIC	10,327.85	8,296.85	8,299.74	8,296.96

Note. Weighted data. OR, Odds Ratio; CI, Confidence Interval; Ref., Reference Category; GISD, German Index of Socioeconomic Deprivation; IADL, Instrumental Activities of Daily Living; BIC, Bayesian Information Criterion; AIC, Akaike Information Criterion. *p < 0.05; **p < 0.01; ***p < 0.001

In adjusted Model 2, the effect of all independent variables with exception of place attachment was reduced. The effect of GISD became statistically insignificant (OR = 0.97; 95% CI = 0.64–1.48). Worsening building condition were related to reduced odds to report good self-rated health (OR = 0.81; 95% CI = 0.73–0.91). Improved walkability (OR = 1.19; 95% CI = 1.10–1.28), higher attachment with the place (OR = 1.14; 95% CI = 1.07–1.20), and higher social cohesion (OR = 1.15; 95% CI = 1.08–1.21), were all associated with increased odds of experiencing good self-rated health.

The inclusion of interaction terms for indicators of socioeconomic (GISD) and physical neighborhood context (building condition, walkability) with place attachment (Model 3a) and social cohesion (Model 3b) revealed no statistically significant associations. The effect of social cohesion on self-rated health slightly decreased (OR = 1.14; 95% CI = 1.08–1.21) in Model 3a and Model 3b. The effect of place attachment on self-rated health declined in Model 3b and became insignificant (OR = 1.08; 95% CI = 0.93–1.26). The estimates of all other independent variables remained at the level of Model 2. For further details, please refer to Table 2. Unstandardised regression coefficients with standard errors are reported in Supplement Table S2.

## Discussion

The findings of the current study indicate that especially social and physical aspects of neighborhood context are associated with self-rated health among very old adults living in Germany. While the effect of the objectively measured indicator of neighborhood socioeconomic context (GISD) on self-rated health disappeared after adjusting for relevant sociodemographic and health characteristics, all perceived characteristics of neighborhood physical (building condition, walkability) and social context (social cohesion, place attachment) remained significant predictors of self-rated health among very older adults in the adjusted model. Nevertheless, the hypothesis assuming that the effect of neighborhood socioeconomic and physical context on self-rated health differ according to available neighborhood social recources could not be confirmed.

In line with previous studies [12, 13, 15], very old population who trusted neighbors and were close connected with their living environment had higher chance to report good self-rated health. Following the social capital theory [25, 26], social inclusion and cohesion in neighborhoods are important resources that protect against adverse health outcomes, such as frailty [22] or depression [38]. Especially during the COVID-19 pandemic, availability of neighborhood social resources has been identified as a key protective factor. For instance, Sato et al. [38] found that the pre-pandemic the reciprocity of social support within communities buffered the development of depression among the older population during the COVID-19 pandemic.

Consistent with Yun [16] and previous studies focusing on physical activity [18], living in better maintained buildings and neighborhoods with higher walkability were associated with higher chance to experience good self-rated health. Following the key assumption of environmental gerontology [3], the misfit between individual competencies and environment over a longer period can lead to reduced quality of life (e.g., restricted autonomy), which may, in turn, result in health deterioration. Wahl and Oswald [5] highlighted that cognitive and emotional evaluation of physical environment are responsible for remaining in environments that no longer fit to the individual funtional abilities and needs particularly in advanced age. Living environments that are poorly maintained and non-barrier-free have been identified to facilitate risky behavior patterns, such as low physical activity [18], leading to deterioration of physical [39, 40] and mental health [41–43] in the long term. For instance, older adults with functional impairments necessitating walking aids may constraine their outdoor physical activity if there are neglected or narrow footpaths in the immediate living environment. In the long term, the restriction of physical activity can result in the development of frailty [44].

The neighborhood socioeconomic context was not associated with self-rated health in the very old population, after adjusting for relevant controls. Interestingly, this finding is consistent with previous study using much smaller geographical area (census) to define neighborhood [13]. Nevertheless, it can not be ruled out that the lacking effect in the adjusted model is due to the relatively broad definition of neighborhood. Another possible explanation for this finding may be the relevance of individual socioeconomic status. There is a widespread evidence of a socioeconomic health gradient that may persist until the oldest age [45, 46]. Deprived neighborhoods are generally characterized by a higher proportion of residents with low socioeconomic status. Therefore, the insignificant effect of GISD in the adjusted model might be a consequence of holding the individual socioeconomic status and health conditions of very older adults constant. Futher explanation could be atributed to the survival effect in the very old population. It is well documented that people with low socioeconomic status are less likely to reach the very old age [47, 48].

Additional regional characteristics were associated with self-rated health. As expected, residence in East Germany was associated with an increased chance of poor self-rated health compared to residence in West Germany. Similar results have been observed among the younger population living in Germany [49]. These disparities are often attributed to socio-political and economic changes in East Germany after the political unification in 1990, which resulted in high unemployment rates, disrupted occupational careers, and involuntary early retirement. Consequently, the East German population demonstrates a higher frequency of risky behavior patterns (e.g., alcohol consumption or smoking), which may negatively affect health status [49].

The assumption based on the environmental docility hypothesis [27] could not be confirmed in the current study. The link of neighborhood socioeconomic and physical context with self-rated health was not moderated by neighborhood social resources. The evidence shows that social network size reduces as people become older [50]. According to the socioemotional selectivity theory [51], older adults tend to maintain primarily emotionally meaningful social relationships. Thus, the missing moderation effect could indicate that closer social ties (e.g., relationships with relatives or friends) might be more important resources helping very old adults to adjust to or compensate for unfavorable neighborhood conditions than neighborhood social ties.

Since the data collection was conducted during the period with the highest incidence of COVID-19 and associated death rates among the very old population in Germany, it is possible that the responses of the study participants were influenced by this extraordinary event. For example, due to the recommendations of social distancing, very old adults might have restricted their outdoor activities which could have considerable negative impact on their health. Moreover, availability of public green spaces and less crowded living areas were identified to be associated with better self-rated health during the COVID-19 pandemic in population aged 18 or older [52]. Assuming that rural areas have more green spaces and lower population density compared to the urban ones, the current analyses could not confirm this effect in the very old population of Germany.

### Strengths and limitations

The current study used data from the only representative study for the population aged 80 or older living in Germany. The main strength of the D80+ study is the inclusion of hard-to-reach population groups (e.g., individuals with considerable health limitations) in the study sample, through the use of proxy interviews. Additionally, the association between neighborhood context and self-rated health in older adults was examined for the first time in Germany using a nationwide representative sample. Moreover, as mentioned above, only a few previous studies have considered simultaneous effects of different neighborhood contexts (social, physical, and socioeconomic neighborhood context) on self-rated health in older populations.

There are also certain limitations to be considered when interpreting the results. First, due to the cross-sectional design of D80+, no causal effects or effects of the COVID-19 pandemic can be identified. Second, owing to the pandemic-related regulations of social distancing, written questionnaires and computer-assisted telephone interviews were conducted, instead of computer-assisted personal interviews. Consequently, the responses to the written questionnaires showed higher rates of missing values. Nevertheless, conducting the analyses with a fully imputed dataset revealed comparable findings. Third, due to the modification of survey mode, certain topics (e.g., duration of residence) were collected only in additional telephone interviews conducted with 3,233 individuals [53]. Since the availability of a telephone number was a key criterion for the participation in additional interviews, this subsample should be considered in separate analyses. Finally, the smallest available nationwide geographical area for the objective regional data was a municipality, which is, in most cases, much larger area than what could be considered as a neighborhood. Future research should include objective regional characteristics at lower geographical levels, such as local districts or streets.

## Conclusions

The findings suggest that different aspects of neighborhood context are associated with self-rated health status among very older adults living in Germany. In particular, positive evaluations of physical neighborhood context (building condition and walkability), high neighborhood social cohesion and close connection to the neighborhood might help to protect very old population against poor self-rated health. Further longitudinal studies are needed to examine whether improvements of the neighborhood environment positively influence health trajectories in very old age considering various aspects of neighborhood context as well as the interplay between them.

### Electronic supplementary material

Below is the link to the electronic supplementary material.

Supplementary Material 1

## Data Availability

The D80+ dataset generated and/or analyzed during the current study is available from the German Center of Gerontology research data repository [54]. The INKAR dataset is available from the Federal Institute for Research on Building, Urban Affairs and Spatial Development [55]. Additionally, the datasets used and/or analyzed in the current study available from the corresponding author on reasonable request.

## Abbreviations

AIC: Akaike information criterion
BIC: Bayesian information criterion
CI: Confidence Interval
D80+: Old Age in Germany
INKAR: Indicators and Maps on Spatial and Urban Monitoring
GISD: German Index of Socioeconomic Deprivation
IADL: Instrumental Activities of Daily Living
ISCED: International Standard Classification of Education
OR: Odds Ratio
SD: Standard Deviation
