# Supplementary material for: Impact of neighborhood context on self-rated health among very old adults living in Germany: a cross-sectional representative study
Source: BMC Geriatr. 2024 Jul 5;24:581. doi: 10.1186/s12877-024-05175-y (PMC11227241; doi:10.1186/s12877-024-05175-y)
Supplement: Supplementary file 1 — Supplementary Material 1 [file 12877_2024_5175_MOESM1_ESM.docx]

**Supplementary materials**

Table S1 Bivariate correlations of included variables (N=8,066)

Table S2 Results of logistic regression analyses predicting the likelihood of reporting good self-rated health in very old population in Germany

(Unstandardised regression coefficients with standard errors; original data; N=8,066)

Table S3 Results of logistic regression analyses predicting the likelihood of reporting good self-rated health in very old population in Germany

(Unstandardised regression coefficients with standard errors; fully imputed data; N=10,578)

Table S1 Bivariate correlations of all included variables (*N*=8,066)

|  | 1. | 2. | 3. | 4. | 5. | 6. | 7. | 8. | 9. | 10. | 11. | 12. | 13. | 14. | 15. | 16. |
| --- | --- | --- | --- | --- | --- | --- | --- | --- | --- | --- | --- | --- | --- | --- | --- | --- |
| 1. Self-rated health | - |  |  |  |  |  |  |  |  |  |  |  |  |  |  |  |
| 2. Place attachment | 0.064 | - |  |  |  |  |  |  |  |  |  |  |  |  |  |  |
| 3. Social cohesion | 0.152 | 0.253 | - |  |  |  |  |  |  |  |  |  |  |  |  |  |
| 4. Building condition | -0.136 | -0.038 | -0.151 | - |  |  |  |  |  |  |  |  |  |  |  |  |
| 5. Walkability | 0.172 | 0.026 | 0.162 | -0.264 | - |  |  |  |  |  |  |  |  |  |  |  |
| 6. GISD | -0.045 | 0.017 | -0.043 | 0.034 | -0.038 | - |  |  |  |  |  |  |  |  |  |  |
| 7. Age | -0.145 | -0.027 | -0.054 | 0.032 | -0.054 | 0.008 | - |  |  |  |  |  |  |  |  |  |
| 8. Sex | 0.089 | -0.043 | 0.022 | -0.051 | 0.049 | -0.008 | -0.121 | - |  |  |  |  |  |  |  |  |
| 9. Living alone | -0.066 | -0.038 | -0.087 | 0.052 | -0.003 | 0.014 | 0.257 | -0.353 | - |  |  |  |  |  |  |  |
| 10. Type of residence | -0.131 | -0.136 | -0.187 | -0.043 | 0.008 | 0.013 | 0.311 | -0.103 | 0.295 | - |  |  |  |  |  |  |
| 11. Migration background | -0.008 | -0.011 | -0.034 | 0.032 | 0.008 | 0.028 | 0.008 | -0.027 | 0.001 | 0.000 | - |  |  |  |  |  |
| 12. Education (middle) | -0.010 | -0.018 | 0.022 | -0.016 | 0.041 | -0.006 | -0.033 | -0.047 | 0.013 | 0.021 | -0.055 | - |  |  |  |  |
| 13. Education (high) | 0.076 | 0.003 | 0.069 | -0.062 | 0.028 | 0.004 | -0.110 | 0.338 | -0.155 | -0.076 | -0.004 | -0.609 | - |  |  |  |
| 14. Region of residence | -0.038 | 0.004 | -0.055 | -0.005 | -0.002 | 0.381 | -0.034 | 0.011 | -0.006 | -0.022 | 0.074 | -0.041 | 0.175 | - |  |  |
| 15. Municipality type | -0.011 | 0.035 | 0.032 | 0.028 | -0.114 | 0.092 | -0.025 | 0.000 | -0.032 | 0.001 | -0.019 | -0.048 | -0.001 | 0.011 | - |  |
| 16. IADL | 0.430 | 0.020 | 0.158 | -0.097 | 0.180 | -0.039 | -0.450 | 0.173 | -0.187 | -0.509 | -0.020 | 0.012 | 0.163 | 0.004 | -0.039 | - |
| 17. Number of chronic diseases | -0.393 | -0.037 | -0.114 | 0.109 | -0.128 | 0.059 | 0.115 | -0.117 | 0.106 | 0.115 | 0.007 | -0.017 | -0.059 | 0.052 | 0.020 | -0.319 |

Note. Weighted data. GISD, German Index of Socioeconomic Deprivation; IADL, Instrumental Activities of Daily Living

Table S2 Results of logistic regression analyses predicting the likelihood of reporting good self-rated health in very old population in Germany

(Unstandardised regression coefficients with standard errors; original data; *N*=8,066)

| *Predictors* | *Model 1* | *Model 2* | *Model 3a* | *Model 3b* |  |
| --- | --- | --- | --- | --- | --- |
|  | Unstand. Est. (S.E.) | Unstand. Est. (S.E.) | Unstand. Est. (S.E.) | Unstand. Est. (S.E.) |  |
| *Independent variables* | | | | |  |
| Place attachment | 0.07 (0.03)* | 0.13 (0.03)*** | 0.13 (0.03)*** | 0.08 (0.08) |  |
| Social cohesion | 0.21 (0.02)*** | 0.14 (0.03)*** | 0.13 (0.03)*** | 0.13 (0.03)*** |  |
| Building condition | -0.29 (0.05)*** | -0.21 (0.06)*** | -0.20 (0.06)** | -0.21 (0.06)*** |  |
| Walkability | 0.33 (0.03)*** | 0.17 (0.04)*** | 0.18 (0.04)*** | 0.17 (0.04)*** |  |
| GISD | -0.43 (0.16)** | -0.03 (0.22) | -0.02 (0.21) | -0.04 (0.22) |  |
| *Control variables* | | | | |  |
| Age (years) |  | 0.02 (0.01)* | 0.02 (0.01)* | 0.02 (0.01)* |  |
| Sex (Ref. female) |  | 0.04 (0.07) | 0.04 (0.07) | 0.03 (0.07) |  |
| Living alone (Ref. no) |  | 0.04 (0.07) | 0.04 (0.07) | 0.04 (0.07) |  |
| Type of residence (Ref. private household) |  | 0.89 (0.17)*** | 0.89 (0.17)*** | 0.91 (0.17)*** |  |
| Migration background (Ref. no) |  | 0.04 (0.08) | 0.05 (0.08) | 0.04 (0.08) |  |
| Education (Ref. low) |  |  |  |  |  |
| middle |  | -0.15 (0.09) | -0.15 (0.09) | -0.15 (0.09) |  |
| high |  | -0.07 (0.10) | -0.07 (0.10) | -0.07 (0.10) |  |
| Region of residence (Ref. West Germany) |  | -0.19 (0.09)* | -0.19 (0.09)* | -0.19 (0.09)* |  |
| Municipality type (Ref. urban) |  | 0.13 (0.11) | 0.13 (0.11) | 0.13 (0.11) |  |
| IADL |  | 0.47 (0.06)*** | 0.47 (0.06)*** | 0.47 (0.06)*** |  |
| Number of chronic diseases |  | -0.27 (0.01)*** | -0.27 (0.01)*** | -0.27 (0.01)*** |  |
| *Interaction terms* | | | | |  |
| Building condition x place attachement |  |  |  | 0.04 (0.05) |  |
| Walkability x place attachement |  |  |  | -0.06 (0.04) |  |
| GISD x place attachement |  |  |  | -0.11 (0.17) |  |
| Building condition x social cohesion |  |  | 0.06 (0.05) |  |  |
| Walkability x social cohesion |  |  | 0.01 (0.03) |  |  |
| GISD x social cohesion |  |  | 0.14 (0.17) |  |  |
| *Model fit* | | | | |  |
| BIC | 10,369.82 | 8,415.77 | 8,439.65 | 8,436.87 |  |
| AIC | 10,327.85 | 8,296.85 | 8,299.74 | 8,296.96 |  |
| Note. Weighted data. SD, standard deviation; GISD, German Index of Socioeconomic Deprivation; IADL, Instrumental Activities of Daily Living; BIC, Bayesian Information Criterion; AIC, Akaike Information Criterion. *p<0.05; **p<0.01; ***p<0.001 | | | | | |

Table S3 Results of logistic regression analyses predicting the likelihood of reporting good self-rated health in very old population in Germany

(Unstandardised regression coefficients with standard errors; fully imputed data^m^; *N*=10,578)

| *Predictors* | *Model 1* | *Model 2* | *Model 3a* | *Model 3b* |  |
| --- | --- | --- | --- | --- | --- |
|  | Unstand. Est. (S.E.) | Unstand. Est. (S.E.) | Unstand. Est. (S.E.) | Unstand. Est. (S.E.) |  |
| *Independent variables* | | | | |  |
| Place attachment | 0.07 (0.02)** | 0.12 (0.03)*** | 0.12 (0.03)*** | 0.07 (0.07) |  |
| Social cohesion | 0.22 (0.02)*** | 0.15 (0.02)*** | 0.15 (0.03)*** | 0.15 (0.32)*** |  |
| Building condition | -0.30 (0.04)*** | -0.22 (0.05)*** | -0.21 (0.05)*** | -0.22 (0.05)*** |  |
| Walkability | 0.33 (0.03)** | 0.18 (0.03)*** | 0.19 (0.03)*** | 0.18 (0.03)*** |  |
| GISD | -0.43 (0.14)** | -0.03 (0.22) | -0.02 (0.19) | -0.00 (0.19) |  |
| *Control variables* | | | | |  |
| Age (years) |  | 0.02 (0.01)* | 0.02 (0.01)* | 0.02 (0.01)* |  |
| Sex (Ref. female) |  | 0.00 (0.06) | 0.00 (0.06) | 0.00 (0.06) |  |
| Living alone (Ref. no) |  | 0.01 (0.06) | 0.01 (0.06) | 0.00 (0.06) |  |
| Type of residence (Ref. private household) |  | 0.76 (0.15)*** | 0.76 (0.15)*** | 0.77 (0.15)*** |  |
| Migration background (Ref. no) |  | 0.12 (0.07) | 0.12 (0.07) | 0.12 (0.07) |  |
| Education (Ref. low) |  |  |  |  |  |
| middle |  | -0.10 (0.08) | -0.10 (0.08) | -0.10 (0.08) |  |
| high |  | 0.00 (0.10) | 0.00 (0.10) | 0.00 (0.09) |  |
| Region of residence (Ref. West Germany) |  | -0.22 (0.09)** | -0.22 (0.08)** | -0.22 (0.08)** |  |
| Municipality type (Ref. urban) |  | 0.17 (0.10) | 0.17 (0.10) | 0.16 (0.10) |  |
| IADL |  | 1.38 (0.06)*** | 1.38 (0.06)*** | 1.37 (0.06)*** |  |
| Number of chronic diseases |  | -0.27 (0.01)*** | -0.27 (0.01)*** | -0.27 (0.01)*** |  |
| *Interaction terms* | | | | |  |
| Building condition x place attachement |  |  |  | 0.03 (0.05) |  |
| Walkability x place attachement |  |  |  | -0.04 (0.03) |  |
| GISD x place attachement |  |  |  | -0.20 (0.15) |  |
| Building condition x social cohesion |  |  | 0.04 (0.05) |  |  |
| Walkability x social cohesion |  |  | 0.01 (0.03) |  |  |
| GISD x social cohesion |  |  | 0.05 (0.14) |  |  |
| *Model fit* | | | | |  |
| BIC^n^ | 13,575.63 | 11,100.15 | 11,126.43 | 11,121.40 |  |
| AIC^n^ | 13,532.03 | 10,976.62 | 10,981.09 | 10,976.07 |  |
| Note. Weighted data. SD, standard deviation; GISD, German Index of Socioeconomic Deprivation; IADL, Instrumental Activities of Daily Living; BIC, Bayesian Information Criterion; AIC, Akaike Information Criterion. *p<0.05; **p<0.01; ***p<0.001  ^m^Multiple imputations were used to replace the missing data pertaining to all the variables included in the analyses. Household size (living alone) and walkability had the highest missing data rates (4.6% and 4.1%, respectively). In addition to all variables included in the analyses, two auxiliary variables (correlated with self-rated health) were considered in multiple imputations: survey mode (written questionnaire/telephone interview) and self-reported deterioration of health status during the COVID-19 pandemic (no/yes). Twenty datasets were generated for this study.  ^n^Values of BIC and AIC are based on average values of the 20 imputed datasets. | | | | | |
